# Repair and remodeling of the mandibular head of the condylar process in four immature dogs

**DOI:** 10.3389/fvets.2023.1288938

**Published:** 2023-11-08

**Authors:** Christopher P. Sauvé, Nadine Fiani, Santiago Peralta, David C. Hatcher, Boaz Arzi

**Affiliations:** ^1^Pulse Veterinary Specialists and Emergency, Sherwood Park, AB, Canada; ^2^Department of Clinical Sciences, College of Veterinary Medicine, Cornell University, Ithaca, NY, United States; ^3^Department of Surgical and Radiological Sciences, University of California, Davis, Davis, CA, United States; ^4^Veterinary Institute for Regenerative Cures, School of Veterinary Medicine, University of California, Davis, Davis, CA, United States

**Keywords:** temporomandibular joint, TMJ, repair, remodeling, regeneration, canine, condylar process, computed tomography

## Abstract

Spontaneous repair and remodeling of the mandibular head of the condylar process is a rarely reported outcome following condylectomy. This clinical report describes the spontaneous repair and subsequent remodeling of the mandibular head of the condylar process in four immature dogs that sustained traumatic injuries, necessitating surgical intervention through arthroplasty via partial or complete condylectomy, or caudal mandibulectomy. In subsequent evaluations, it was observed that all dogs exhibited clinically functional TMJs, as evidenced by an appropriate range of motion. These findings were corroborated by the owners’ reports of the patient’s normal eating and drinking abilities. Conventional and cone-beam computed tomography studies demonstrated the repair and remodeling of the osseous tissues of the mandibular head of the condylar process. Histopathology was unavailable to assess the novel tissues. No evidence of intraarticular or extraarticular ankylosis or osteoarthritic changes was observed.

## 1. Introduction

The temporomandibular joint (TMJ) is a synovial joint composed of the mandibular head of the condylar process and the mandibular fossa of the squamous temporal bone (1, 2). In the canine species, morphological and morphometric descriptions of the TMJ have identified substantial anatomical variations in the shape of both the mandibular head of the condylar process and the retroarticular process, as well as variations in the depth of the mandibular fossa and the congruity between the joint’s bones (2, 3). Furthermore, specific dog breeds may also show variations in the shape of the mandibular head of the condylar process and the condylar process within their breed (3).

The TMJ comprises two separate, non-communicating compartments: the dorsal (temporal) and ventral (mandibular), separated by an intraarticular fibrocartilaginous disc (1). The articular surfaces of the TMJ consists of fibrocartilage, containing both Type I and II collagen, which contrasts with hyaline cartilage that is predominately composed of type II collagen (4). The interaction between the fibrocartilaginous disk and the fibrocartilage articular surfaces aligns the tissues, facilitating smooth movement, reducing friction, and enabling them to withstand high shearing and compressive forces (4, 5). A fibrous capsule surrounds the TMJ, attaching circumferentially to the articular disc, with a lateral ligament providing support (5).

Proper TMJ function is crucial for overall well-being, as it plays an integral role in activities such as eating, drinking, grooming, vocalization, thermoregulation, and protection (1). Therefore, TMJ health degradation may lead to impaired function, pain, tissue degeneration, and disc damage (5, 6).

In dogs, the TMJ functions as a hinge-like articulation, with the mandibular head of the condylar process rotating within the joint to enable mouth opening and closing (1, 5, 7). The joint has limited lateral movement capacity (laterotrusion) (5, 7). Furthermore, it has been reported that approximately 50% of the studied dogs exhibited the capability of translation of the mandibular head of the condylar process during mouth opening. Translation involves the mandibular head of the condylar process sliding forward and downward within the glenoid fossa when the mouth is opened (8).

Accurate diagnosis of TMJ diseases relies on advanced diagnostic imaging. In human medicine, computed tomography (CT) and magnetic resonance imaging (MRI) are the preferred imaging modalities for evaluating both hard and soft tissues of the TMJ (5, 9–11). In veterinary medicine, CT is currently the most widely used diagnostic modalities for assessing osseous lesions and spatial relationships among the TMJ’s bony structures (5). Although conventional skull radiographs offer high spatial resolution, they provide an inferior diagnostic yield compared to CT or CBCT when assessing the structures of the TMJ due to numerous superimposing osseous structures (5, 10, 12).

In veterinary medicine, condylectomy and gap arthroplasty are the most common excisional surgical interventions involving the TMJ. Gap arthroplasty, a more complex procedure, includes zygomectomy, coronoidectomy, condylectomy, and removal of the mandibular fossa of the temporal bone (13, 14). Gap arthroplasty is primarily indicated for intra- or extraarticular ankylosis (13, 14).

Condylectomy is primarily indicated for severe traumatic injuries to the mandibular head of the condylar process (e.g., comminuted fractures with displacement or avulsion) where comfortable opening and closing of the mouth is not possible, the inability to reduce a luxated condylar process using closed or open reduction techniques, neoplasia, debilitating osteoarthritis, and intra- or extraarticular ankylosis (15, 16). Patients may experience adverse functional and anatomical outcomes following a condylectomy or gap arthroplasty, often experiencing mandibular drift with subsequent malocclusion, leading to trauma to hard and soft tissues, functional stress on the contralateral TMJ resulting in degenerative changes and atrophy of the masticatory muscles (16).

Wound healing is achieved through either repair or regeneration. Repair, or incomplete regeneration, describes the outcome of wound healing where tissues do not maintain the same structure and function as the original tissues, often involving the formation of a fibrous, connective-tissue scar (17). Regeneration, or complete regeneration, describes the outcome of wound healing where tissues maintain the same structure and function as the original tissues (17). Specifically, bone regeneration and repair serves as a process aimed at restoring the integrity of the skeletal framework and restoring function (18). The process of bone healing involves osteoinduction, osteoconduction, various cell types, and molecular signaling pathways (18). Fracture healing is the most common clinical scenario in which bone regeneration and remodeling take place (18). In comparison to long bones, the mandible displays enhanced osteogenic potential due to its elevated angiogenic capabilities and enhanced bone-forming gene expression in mandibular bone marrow stromal cells (BMSC), in contrast to femur-derived BMSC (19). The TMJ disc is believed to play a significant role in regeneration of the mandibular head of the condylar process, and its preservation during condylectomy is appears crucial to prevent intraarticular fibrous ankylosis (20). Limited reports are available describing the repair or regeneration of the mandibular head following condylectomy in humans (21–26) or the mandibular head of the condylar process in animals (27–31).

Regeneration of the mandibular head of the condylar process would involve restoration of the osseous and fibrocartilaginous tissues, as well as maintaining a functional intraarticular fibrocartilaginous disc, such that, both morphology and functionality are completely restored to previous states. Confirming complete anatomical tissue regeneration after excision is not possible without histopathological examination. In the absence of histopathological assessment, this case series relies on CT and CBCT images to demonstrate the regrowth of the mandibular head of the condylar process, as well as maintenance of the TMJ space. The CT/CBCT images demonstrate a condyle-like shaped bony structure with irregular margins. Therefore, in the absence of complete restoration of the morphologic and histologic shape of the mandibular head of the condylar process, repair rather than regeneration best describes the clinical outcome. Modeling and remodeling processes were involved in the repair process of the arthroplasty sites, resulting in regrowth of the osseous structures. Modeling of the mandibular head of the condylar process involves an uncoupled osteoblastic and osteoclastic function, allowing the bone to enlarge and drift in three-dimensional space (32). Remodeling of the mandibular head of the condylar process involves a coupled osteoblast–osteoclast function within the bone’s internal anatomy to allow it to maintain its structure (32).

Hence, we present a case series of four immature dogs who underwent arthroplasty involving condylectomy or caudal mandibulectomy following traumatic TMJ injury. Clinical assessment included examination of the range of motion and owners’ perception of the patients comfort and function. Due to the clinical nature of this report, histological analysis of the tissues was not performed, limiting the assessment of the repaired tissues and maintained TMJ disc. Confirmed with CT or CBCT, all four dogs displayed spontaneous repair of osseous structures resembling a condylar process with a well-defined mandibular head. No evidence of intra- or extraarticular ankylosis was observed. Common features of these osseous tissues were compared to determine their presence or absence (Table 1).

**Table 1:** Common features of the repaired and remodeled osseous tissues in four cases presented in this case-series.

	Case 1	Case 2	Case 3	Case 4
Features				
Repaired and remodeled mandibular head of the condylar process	Yes	Yes	Yes	Yes
Normal internal mandibular head of the condylar process architecture	Yes	No	Yes	Yes
Normal mandibular head size	Small	Small	Small	Yes
Smooth mandibular head of the condylar process articular surface	Yes	Yes	Yes	Yes
Ventral positioning of TMJ	Yes	No	No	No
Rostral positioning of TMJ	Yes	No	Yes	No
Vertically shortened Mid ramus	Yes	Yes	Yes	Yes
Vertically shortened molar part of mandible	Yes	No	Yes	Yes
Medial position of mandible	Yes	No	Yes	Yes
Midline shift of mandible	Yes	No	Yes	No
Occlusal plane shift	Yes	No	Yes	No

## 2. Case reports

### 2.1. Case 1

**Figure 1:**
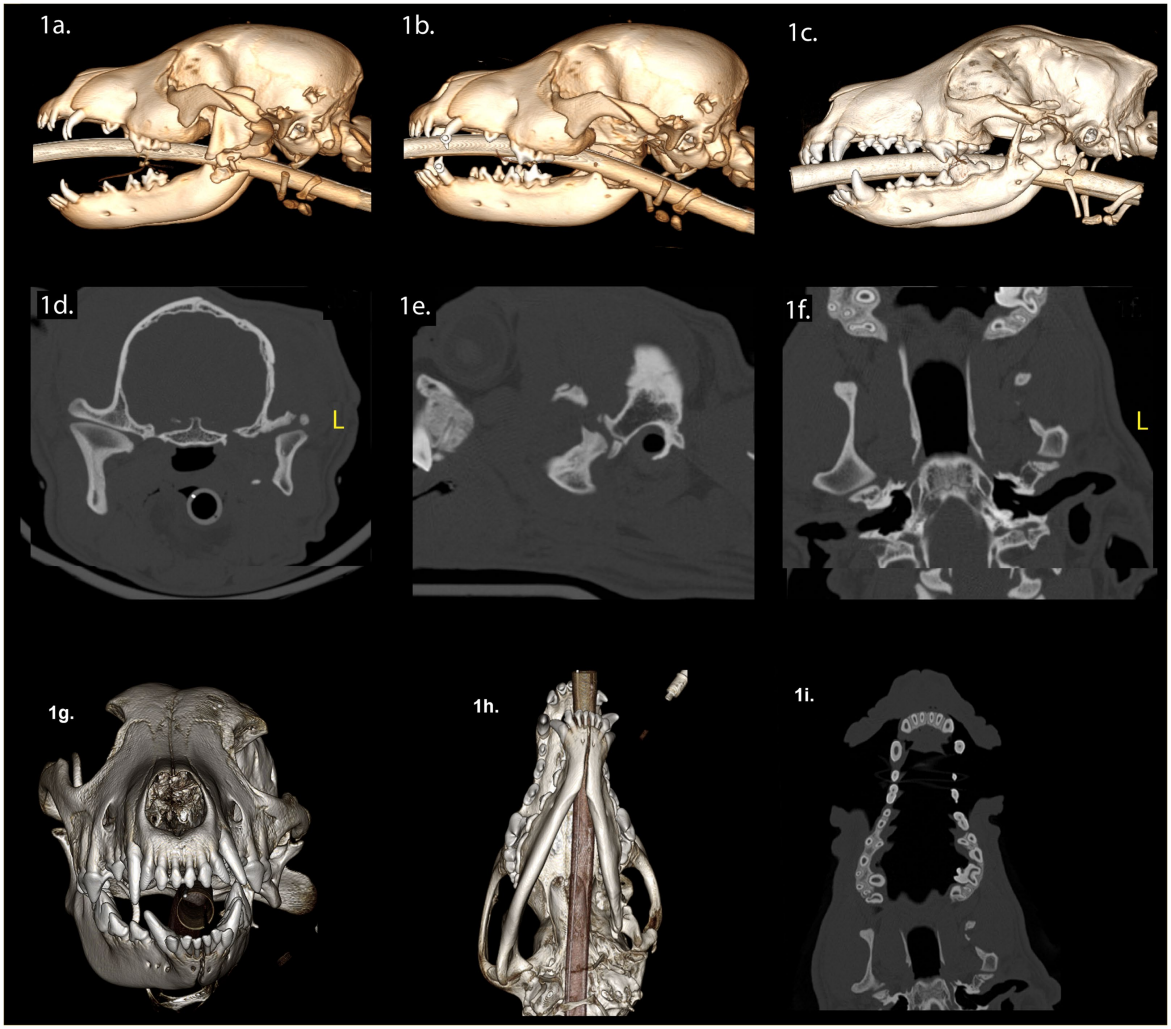
Panels **(A–I)** represent the diagnostic images associated with case 1. **(A)** Perioperative CT, 3-D reconstruction of the skull of the patient demonstrates comminuted fractures of the left ramus and the coronoid process. **(B)** Immediate postoperative CT, 3-D reconstruction of the skull of the patient demonstrates the left caudal mandibulectomy. **(C)** Twenty-week postoperative CT, 3-D reconstruction image of the skull demonstrates the newly modeled and remodeled left mandibular head of the condylar process, the condylar process, and a portion of the coronoid process. **(D–F)** Twenty-week postoperative CT demonstrates the transverse, parasagittal and dorsal planes of the skull at the level of the TMJ, highlighting the newly formed left mandibular head of the condylar process and the condylar process. The transverse dimensions of the left mandibular head of the condylar process and the condylar process are comparatively smaller in relation to contralateral structures. The medial pole of the mandibular head is not evident. The left TMJ is positioned rostrally on the cranial base when compared to the right side. The articular portion of the left glenoid fossa is rostrally positioned on the cranial base. Panel **(G)** (rostral view) and **(H)** (ventral view) are volume rendered images. There is asymmetry of the mandibles with the left mandible being smaller than the right mandible (transverse, vertical, and rostrocaudal). The osseous and dental midlines of the mandible are shifted to the left. The occlusal plane is elevated on the left side. **(I)** The shape of the maxilla has not significantly changed when comparing the preoperative study and the 20-week postoperative study.

A 3-month-old intact female mixed breed dog was presented with maxillofacial injuries following a dog bite. Conventional CT imaging of the head (Figure 1) revealed several injuries: (A) A left condylar process fracture with lateral displacement. (B) A comminuted fracture of the left ramus with medial displacement of a bone fragment containing the angular process. (C) An oblique fracture separating the coronoid process from the ramus. (D) Avulsion of the developing mineralized tooth structures of the left mandibular second molar tooth. (E) fracture of the left caudal maxilla. (F) A temporal bone fracture resulting in lateral displacement of the zygomatic arch. (G) A separation of the zygomaticotemporal suture and (H) a fracture of the temporal process of the zygomatic bone.

The surgical intervention involved excision of the left caudal mandible, including the displaced mandibular head of the condylar process, while preserving the TMJ disc (Figure 1B). The left mandibular second molar was extracted (Figure 1B). For the purpose of promoting functional occlusion, orthodontic buttons with elastomeric chains were placed on the deciduous canine teeth (Figure 1B). These remained in place until the elastics dislodged, failed, or the deciduous teeth exfoliated. Following surgical treatment, we conducted a conventional CT scan of the head (Figure 1B). Upon discharge, the dog received a 4-day course of meloxicam (0.1 mg/kg once daily), a 5-day course of gabapentin (10 mg/kg twice or three times daily), and 7-day course of clindamycin (5 mg/kg twice daily).

Twenty weeks post-surgery, we observed a class 4 malocclusion with mandibular deviation to the left during conscious oral examination (33). The patient displayed near-normal range of motion and could open and close the mouth without limitations. Conventional CT imaging of the head (Figures 1C–I) revealed several findings: (A) Shifting of the mandible to the left and a shorter rostrocaudal dimension on the left mandible. (B) Reduced left mandibular lateral development. (C) Decreased vertical dimensions on the left, including the ramus, condylar process, and the mandibular body region (molar, premolar, canine, and incisor). (D) Evidence of bone bridging between the ramus, condylar process, and the coronoid process. (E) A lateral step deformity extending from the ramus to the condylar process and the angular process, resulting in lateral cantilevering of the condylar process. (F) Depression of the left orbit compared to the right. (G) Regrowth of the left mandibular head of the condylar process with smaller overall dimensions, particularly the medial pole, while the lateral half maintained a relatively normal shape. (H) Both the mandibular head of the condylar process and the condylar process exhibited an outer cortex and internal cancellous bone. (I) Non-union of the fracture of the left zygomatic arch. (J) The left TMJ was positioned rostroventrally on the skull base compared to the right, with an unattached retroarticular process. Finally, while the articular surface of the left mandibular head appeared round and smooth, the left TMJ space exhibited non-uniformity compared to the right.

### 2.2. Case 2

**Figure 2:**
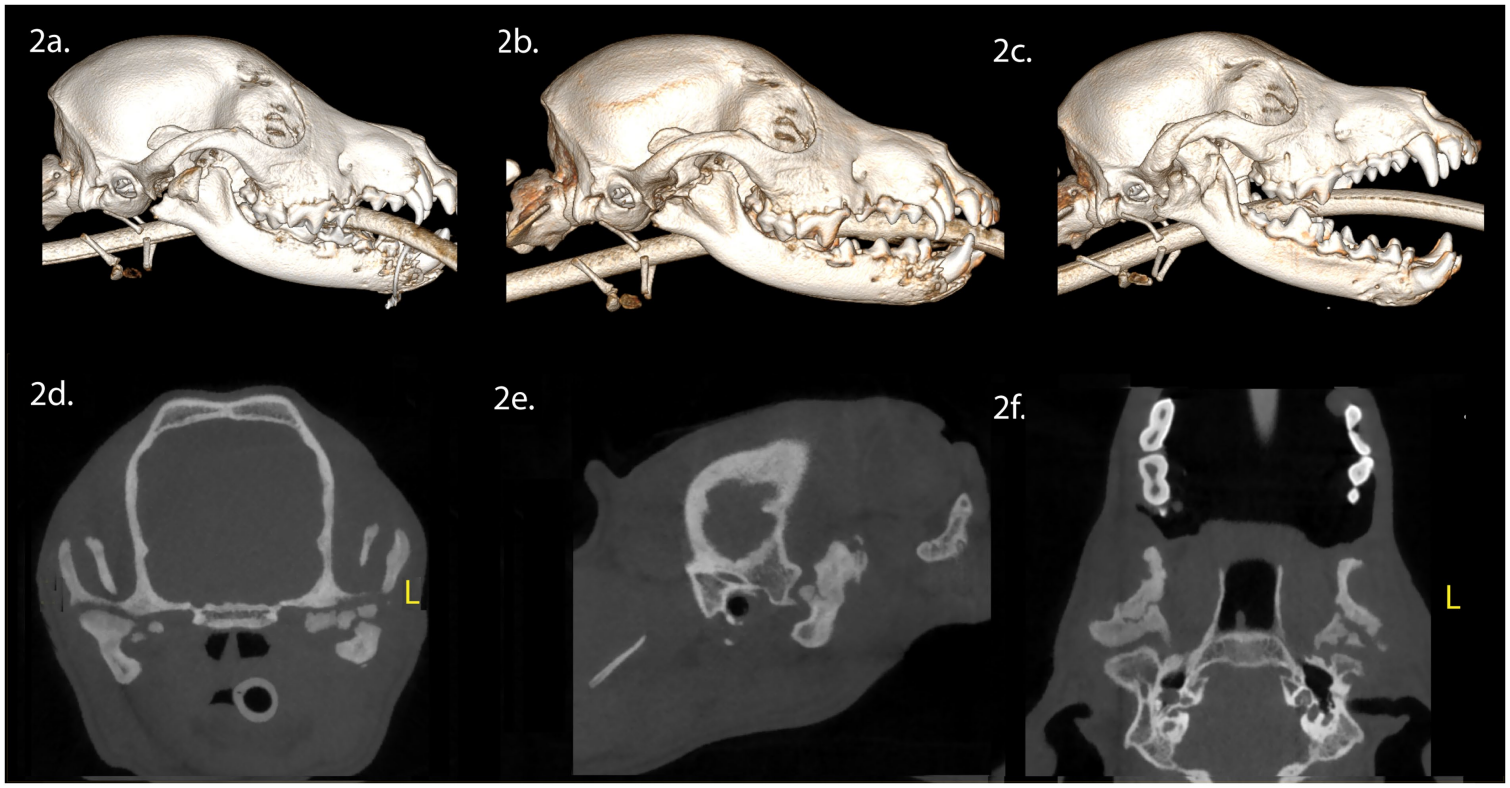
Panels **(A–F)** represent the diagnostic images associated with case 2. **(A)** Perioperative CT, 3-D reconstruction of the skull of the patient demonstrates the comminuted fracture of the right mandibular head of the condylar process, ramus and coronoid process. **(B)** Immediate postoperative CT, 3-D reconstruction of the skull demonstrating the condylectomy. **(C)** Five-week postoperative CT, 3-D reconstruction of the skull demonstrating the newly modeled and remodeled right mandibular condylar process. **(D–F)** Five-week postoperative CT demonstrates the transverse, parasagittal and dorsal plane of the skull at the level of the TMJ, highlighting the right mandibular head of the condylar process and non-union of the left mandibular head of the condylar process.

A 7-month-old intact female Chihuahua mix dog presented with maxillofacial injuries sustained in a vehicular accident. CBCT imaging of the head (Figure 2) revealed several injuries: (A) A right ramus fracture, resulting in lateral condylar process luxation and lateral displacement of the fractured condylar process. (B) A fracture involving both the left and right mandibles, crossing the separated symphysis in a lateroventral direction, involving the alveoli of the left and right permanent mandibular canine teeth and (C) Dorsocaudal luxation of the left TMJ, which included a comminuted fracture of the left mandibular head of the condylar process and a ramus fracture resulting in lateral displacement of the left coronoid process.

Surgical intervention involved a right condylectomy and excision of the displaced mandibular head of the condylar process, while preserving the TMJ disc (Figure 2B). Surgical treatment for the left TMJ was not performed as the injury did not impede opening and closing of the mouth. A postoperative CBCT scan of the head was performed following surgical treatment (Figure 2B). Upon discharge, the patient received a 10-day course of tramadol (6 mg/kg, PO, three times daily), a 5-day course of meloxicam (0.1 mg/kg, PO, once daily), and a 10-day course of amoxicillin clavulanate (15 mg/kg, PO, twice daily).

Five weeks post-surgery, we noted a class 4 malocclusion with rightward mandibular deviation during conscious oral examination. Despite this, the patient displayed near-normal range of motion and was able to open and close the mouth without limitations. CBCT imaging of the head (Figures 2C–F) revealed several findings: (A) Healing of the right coronoid process fracture, which had fused with the ramus. (B) Regrowth of the right mandibular head of the condylar process with a smooth and corticated articular surface, situated in a shallow fossa. (C) A relatively larger left TMJ space compared to the right. (D) Non-union of the untreated left condylar fracture, resulting in irregular positioning of the proximal segment of the mandibular head of the condylar process within the fossa.

### 2.3. Case 3

**Figure 3:**
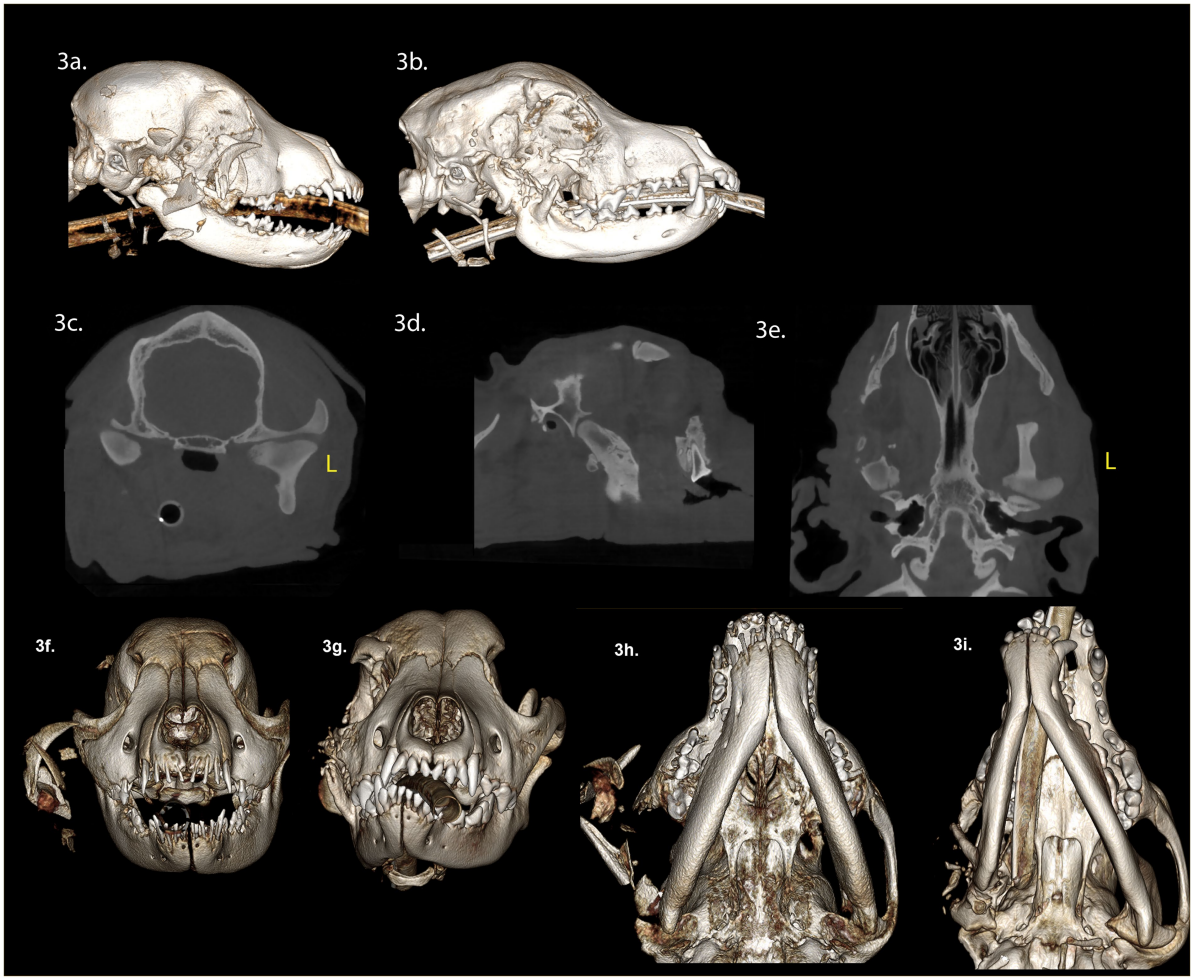
Panels **(A–F)** represent the diagnostic images associated with case 3. **(A)** Perioperative CT, 3-D reconstruction of the skull of the patient demonstrates a comminuted fracture of the right mandibular head of the condylar process, ramus, zygomatic arch, and coronoid process with significant displacement of the fragments. **(B)** Four-week postoperative CT, 3-D reconstruction of the skull demonstrates the newly modeled and remodeled mandibular head of the condylar process and the condylar process. **(C–E)** Four-week postoperative CT demonstrates the transverse, parasagittal and dorsal plane of the skull at the level of the TMJ, highlighting the newly modeled and remodeled right mandibular head of the condylar process, and condylar process. The transverse dimension of the right mandibular head of the condylar process, and condylar process are comparatively smaller in relation to the contralateral structures. The lateral dimensions of the right mandibular head of the condylar process are smaller than the left mandibular head of the condylar process. Panels **(F,G)** are rostral views of the skull pre-surgery and 4 weeks post-surgery. The osseous and dental midlines of the mandible are shifted to the right after 4 weeks. There is a right sided caudal cross-bite and an open bite on the left side. The occlusal plane on the right side is elevated. Panels **(H,I)** are volume rendered images pre- and 4 weeks post-surgery. The rostrocaudal dimensions of the right mandible are comparatively less than the left mandible. The right TMJ is positioned rostrally on the cranial base compared to the left.

A 4-month-old spayed female Staffordshire bull terrier presented with maxillofacial injuries following a dog bite. CBCT scan of the head (Figures 3A,F,H) revealed several injuries: (A) A comminuted right ramus fracture involving the mandibular head of the condylar process and coronoid process, with significant displacement. (B) A comminuted right zygomatic arch fracture, with significant displacement. (C) Soft tissue swelling along the right side of the face, and isolated areas of gas attenuation in the soft tissues lateral to the right maxilla and mandible, indicative of air emphysema in the traumatized tissues.

Surgical intervention involved excising the comminuted fragments of the right zygomatic arch and the caudal mandible, including the condylar process, while preserving the TMJ disc. Postoperatively, a three-dimensional printed soft facemask was applied to stabilize facial structures and provide elastic support to the TMJ. Upon discharge, the patient received a 10-day course of carprofen (3 mg/kg twice daily), a 14-day course of amoxicillin clavulanate (14.5 mg/kg twice daily), and a 5-day course of tramadol (6 mg/kg PO twice of three times daily).

Four weeks post-surgery, we observed a class 4 malocclusion with rostral mandibular deviation to the right during a conscious oral examination. Despite this, the patient demonstrated near-normal range of motion and was able to open and close the mouth without limitations. CBCT imaging of the head (Figures 3B–E,G,I) revealed several findings: (A) Horizontal mandibular drift to the right side. (B) A left caudal open bite. (C) A right maxillary canine crossbite. (D) A step deformity of the right lateral frontal bone. (E) Predominately absent zygomatic process of the right temporal bone and absence of the right coronoid process. (E) Multiple small remaining bone fragments in the soft tissues lateral to the maxilla and mandible. (F) New bone extending from the proximal end of the ramus fracture site to the zygomatic process. (G) The newly formed bone closely resembling the contralateral mandibular head of the condylar process, featuring a well-defined, smooth, nearly convexly rounded shape. (H) The outer cortex of the condylar process was well-defined, with a central region of cancellous bone. (I) The mediolateral dimensions of the right condylar process were smaller than the left condylar process. (J) The circumference of the right condylar process neck was greater than the left condylar process. (K) The margins of the right zygomatic process exhibited focal areas of irregular contour. (L) The caudal wall of the right zygomatic process was positioned rostrally compared to the left zygomatic process. A larger and more variable joint space was observed on the right TMJ compared to the left TMJ. Additionally, appositional bone formation was noted along the ventral border of the mandible, filling the depressions between the ramus and condylar process.

### 2.4. Case 4

**Figure 4:**
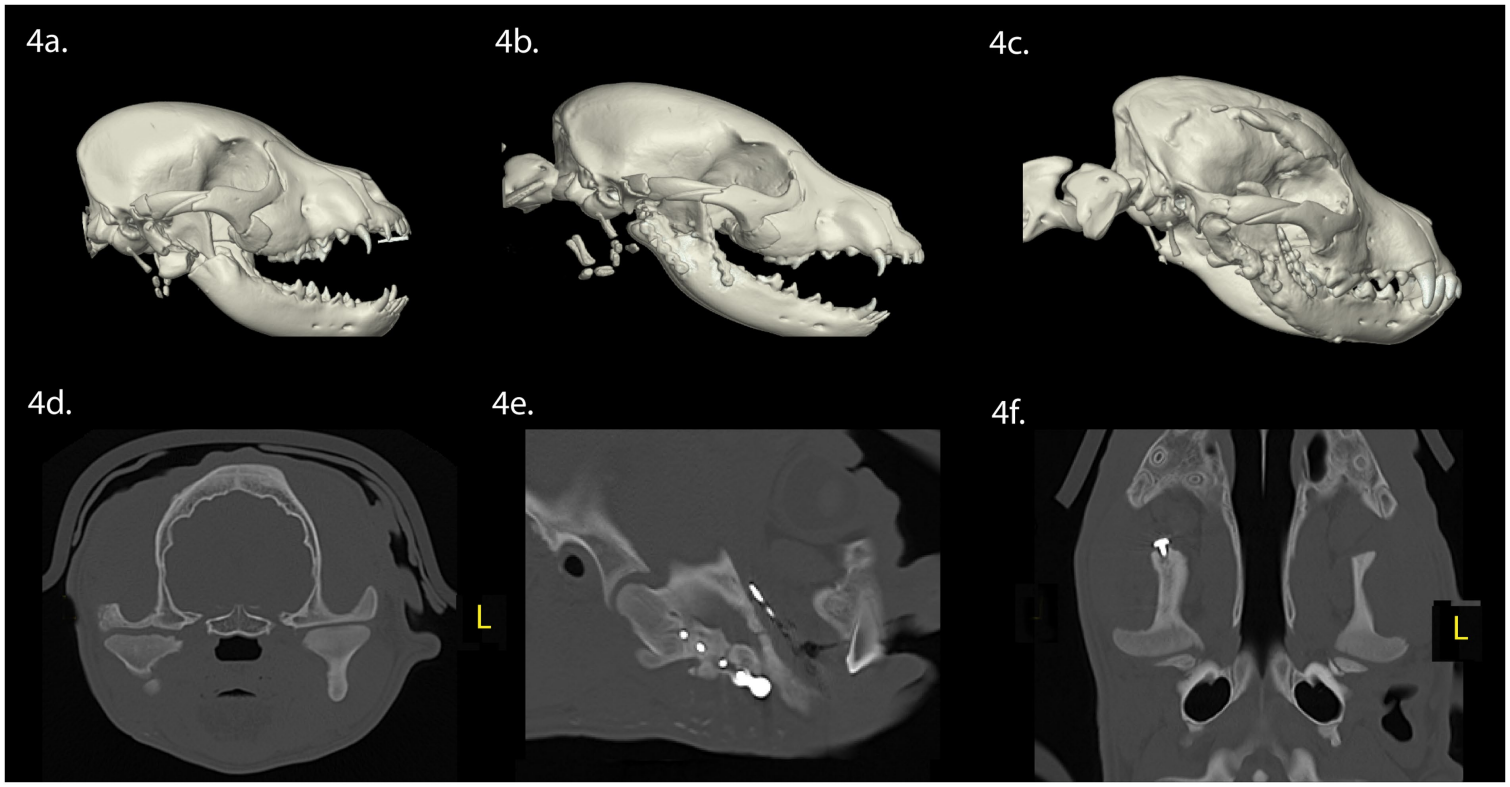
Panels **(A–F)** represent the diagnostic images associated with case 4. **(A)** Perioperative CT, 3-D reconstruction of the skull of the patient demonstrates fractures of the right mandibular head of the condylar process, ramus, and the zygomatic arch. **(B)** Immediate postoperative CT, 3-D reconstruction of the skull demonstrates the removal of the fractured portion of the mandibular head of the condylar process, and internal fixation of the right ramus and coronoid process with a titanium miniplate and locking screw construct. **(C)** Eight-week postoperative CT, 3-D reconstruction of the skull demonstrating the newly modeled and remodeled right mandibular head of the condylar process. **(D–F)** Five-week postoperative CT demonstrates the transverse, parasagittal and dorsal plane of the skull at the level of the TMJ, highlighting the right mandibular head and condylar process. The right and left TMJs are relatively symmetric. There is flattening of the articular surface of the right mandibular head of the condylar process.

A 3-month-old intact male Staffordshire bull terrier dog was presented with maxillofacial injuries sustained from a dog bite. Conventional CT imaging of the head (Figure 4) revealed several injuries: (A) Comminuted fractures of the right mandibular head, condylar process, ramus, and coronoid process. (B) Displacement of the lateral fragment of the right mandibular head in a rostrolateral direction. (C) A segmental, minimally displaced fracture of the right zygomatic arch extending into the squamous portion of the temporal bone.

Surgical intervention involved stabilization of the fractures of the ramus and coronoid process through open reduction and internal fixation using titanium miniplate and screw construct. Retrieval of the highly mobile fragment of the fractured right condylar process was performed, which had been displaced laterally without soft tissue attachment, while preserving the TMJ disc. A postoperative conventional CT of the head was performed following surgical treatment (Figure 4B). Upon discharge, the patient received a 5-day course of meloxicam (0.1 mg/kg once daily), a 14-day course of amoxicillin clavulanate (12.5 mg/kg twice daily), and a 5-day course of tramadol (2.5 mg/kg three times daily).

Eight weeks post-surgery, a conscious examination revealed normal occlusion. The patient demonstrated a near-normal range of motion, with the ability to open and close the mouth without limitations. Conventional CT imaging of the head (Figures 4C–F) revealed several findings: (A) The right ramus and molar regions of the right mandible exhibited greater mediolateral dimensions and thicker lingual and buccal cortices when compared to the left ramus and mandible. (B) Vertical dimensions of the right ramus and mandible were smaller, when compared to the left ramus and mandible. (C) The right angular process was reduced in size, when compared to the left angular process. (D) The right zygomatic process had healed with a minor step deformity. (E) The right coronoid process was aligned normally, indicating successful repair of the fracture segments. (F) Regrowth of the right mandibular head of the condylar process was evident, featuring an outer cortex and inner trabecular bone. The mandibular head displayed a rounded articular surface with focal irregular and flattened regions. (G) The mediolateral dimensions of the right condylar process were comparable with the contralateral side, with the right mandibular condylar process having a greater circumference than the left condylar process. (H) The lateral pole of the right mandibular head exhibited a mild rostral tilt, similar to the left mandibular head. (I) While the right retroarticular surface and condylar process formed a relatively congruent articulation, the TMJ space was wider that the left TMJ. Finally, the right TMJ was positioned rostroventrally compared to the left TMJ.

## 3. Discussion

This case series presents four instances of spontaneous repair of the mandibular head of the condylar process in immature dogs following excision after traumatic injury. In each case, CT and CBCT scans revealed rapid wound healing of the arthroplasty site, involving incomplete regeneration of the osseous tissues through modeling and remodeling of the mandibular head of the condylar process (range: 4–20 weeks post-injury). Most dogs exhibited a well-formed cortical articular surface and trabecular bone pattern within the mandibular head of the condylar process. Functionally, these dogs maintained near-normal range of motion, though class 4 malocclusions were common. CT or CBCT imaging demonstrated variations in skeletal dimensions of the newly formed mandibular osseous tissues compared to the contralateral (untreated) mandible. Importantly, despite severe TMJ trauma and surgical intervention involving the TMJ in these young dogs, neither intraarticular nor extraarticular TMJ ankylosis occurred.

Due to the clinical nature of this case series, histopathology of the newly formed anatomical structures was not conducted. Consequently, the authors cannot provide a histologic description of the repaired tissues, nor confirm the maintenance of the intraarticular fibrocartilaginous disc. However, our study findings are radiographically consistent with a prior report that investigated both radiographic and histologic changes in the mandibular head of the condylar process between 1 and 3 months following unilateral condylectomy (27). In that study, the authors performed histopathologic analysis, which confirmed the regeneration of the anatomical structures, including the mandibular head of the condylar process, the fibrocartilaginous articular surfaces, and the maintenance of the preserved fibrocartilaginous intraarticular disc (27). Furthermore, the study revealed a notable difference in the degree of regeneration between various anatomical locations. Specifically, there was relatively more pronounced regeneration of the mandibular head of the condylar process in medial and rostral aspects compared to lateral and caudal areas (27), indicating increased structural and functional stresses in these regions. Importantly, within the joint space, irregular regenerated articular cartilage was observed, accompanied by central thickening of the TMJ disc (27).

It is believed that immature dogs tend to demonstrate enhanced potential for bone regeneration (27, 34). Beyond age considerations, the regeneration of the mandibular head of the condylar process may involve multiple mechanisms contributing to bone formation following excision. The TMJ disc is believed to play a pivotal role in initiating regeneration of the mandibular head of the condylar process by stimulating periosteal cells (20). These differentiating periosteal cells, collectively referred to as a blastema, are primarily situated in the medial region of the ramus (35). Furthermore, research has demonstrated that in cases where a TMJ disc is absent following condylectomy there is a failure to regenerate the mandibular head of the condylar process, leading to intraarticular fibrous ankylosis (36). Notably, the developmental process of the fetal condylar process shares similarities with the regeneration of the mandibular head of the condylar process (35).

The “diamond concept” of fracture healing is informative when applied to the regeneration of the mandibular head of the condylar process to categorize the numerous factors involved in the regenerative process. This concept suggests that osteoconductive scaffolds, growth factors, osteogenic cells, and the mechanical environment interact synergistically during the repair and regeneration processes (18, 37). During bone regeneration, the regional and intermediate tissues that precede final bone formation, such as connective tissue, cartilage, and woven bone act at the osteoconductive scaffold (18, 37). Although not a comprehensive list, important growth factors involved in stimulating bone regeneration appear to be transforming growth factor beta, bone morphogenetic proteins, fibroblast growth factor, vascular endothelial growth factor, platelet-derived growth factors, and insulin-like growth factor-1 (18, 38, 39). Immunocompetent periosteum including undifferentiated mesenchymal cells, persistent small bone fragments and ostectomy sites appear to serve as primary sources of osteogenic precursor cells (25).

The mechanical environment surrounding the regenerating mandibular head of the condylar process, in conjunction with the TMJ disc and the temporal bone, involves dynamic movement and differs significantly from the process of fracture healing in long bones. It is believed that restoring normal function to the TMJ is crucial for successful regeneration of the mandibular head of the condylar process (28). This believe aligns with Wolff’s law, which posits that mechanical forces applied to bone lead to structural changes (40).

Experimental studies have revealed that, following condylectomy, the soft tissue support provided by the masticatory muscles, along with continued loading of the surgically treated TMJ, may create a conducive environment for regeneration (28). A more contemporary model, Frost’s mechanostat model, considers various factors including bone strength, applied forces, hormonal influences, nutrition, the nervous system, behavior, medications, and environmental factors when assessing bone strain (40). Applying these models to the dynamic process of repair or regeneration of the mandibular head of the condylar process presents challenges. However, once bone repair or regeneration initiates, the ongoing functional utilization of the masticatory apparatus continues to exert stress and strain on the tissues, facilitating dynamic bone modeling and remodeling. This phenomenon aligns with the observative made in the cases presented in this report.

There are notable similarities between regeneration of the mandibular head of the condylar process and the evolutionary development of the TMJ in mammals (41). The development of the TMJ appears to be linked with the preceding evolutionary modifications in the dentition of early mammals (41). These alterations in the dentition imposed added stress on the primary jaw joint, leading to the emergence of supplementary bone structures designed to provide support for the mandible during mastication (41), culminating in the formation of the TMJ.

A serious but rare complication following injury or surgical intervention of the TMJ is intraarticular and extraarticular ankylosis (42). This condition severely limits TMJ range of motion, affecting essential functions such as eating, drinking, grooming, vocalizations, thermoregulation, and protection (1). Untreated, it may lead to asymmetrical mandibular growth and the inability to open the mouth (43). A previous study on 94 immature dogs with craniomaxillofacial trauma found that 36.2% had TMJ articular surface fractures, but none developed intraarticular ankylosis, and 10.3% developed extraarticular ankylosis (44). In our case series of four dogs undergoing condylectomy and caudal mandibulectomy, no patient experienced intraarticular or extraarticular ankylosis, reinforcing the rarity of these complications following TMJ trauma demonstrated in this report.

In summary, this case series provides an illustration of the clinical outcomes and tomographic features of spontaneous repair of the mandibular head of the condylar process following arthroplasty, involving either condylectomy or caudal mandibulectomy, in four immature dogs. The observed capacity for regrowth of excised osseous tissues underscores the need for further research aimed at refining treatment options for TMJ trauma in dogs. While these cases demonstrated favorable outcomes, it is important to acknowledge that repair, or incomplete regeneration of the mandibular head of the condylar process remains an unpredictable and poorly understood phenomenon.

## Data availability statement

The original contributions presented in the study are included in the article/supplementary material, further inquiries can be directed to the corresponding author.

## Ethics statement

Ethical approval was not required for the studies involving animals in accordance with the local legislation and institutional requirements because This case series retrospectively demonstrates changes that occurred in clinical practice. Written informed consent was obtained from the owners for the participation of their animals in this study.

## Author contributions

CS: Conceptualization, Data curation, Resources, Visualization, Writing – original draft, Writing – review & editing, Investigation. NF: Conceptualization, Investigation, Methodology, Resources, Visualization, Writing – review & editing. SP: Conceptualization, Investigation, Methodology, Resources, Visualization, Writing – review & editing. DH: Data curation, Investigation, Resources, Visualization, Writing – review & editing. BA: Conceptualization, Investigation, Methodology, Resources, Supervision, Visualization, Writing – review & editing.
